# Immune Response to CoronaVac and Its Safety in Patients with Type 2 Diabetes Compared with Healthcare Workers

**DOI:** 10.3390/vaccines11030684

**Published:** 2023-03-17

**Authors:** Bothamai Dechates, Thachanun Porntharukchareon, Supamas Sirisreetreerux, Phonthip Therawit, Supanat Worawitchawong, Gaidganok Sornsamdang, Kamonwan Soonklang, Kriangkrai Tawinprai

**Affiliations:** 1 Department of Medicine, Chulabhorn Hospital, Chulabhorn Royal Academy, 906 Thung Song Hong, Lak Si, Bangkok 10210, Thailand; 2 Central Laboratory Center, Chulabhorn Hospital, Chulabhorn Royal Academy, 906 Thung Song Hong, Lak Si, Bangkok 10210, Thailand; 3 Center of Learning and Research in Celebration of HRH Princess Chulabhorn 60th Birthday Anniversary, Chulabhorn Royal Academy, 906 Thung Song Hong, Lak Si, Bangkok 10210, Thailand

**Keywords:** diabetes mellitus, CoronaVac, Sinovac, anti-RBD, immunoglobulin

## Abstract

Background: Vaccines for SARS-CoV-2 have been critical for preventing disease. Previous research showed patients with diabetes have impaired immunity. This study aimed to determine the immunity to coronavirus after CoronaVac by comparing patients with type 2 diabetes (T2D) and healthcare workers (HCW). Materials and methods: A prospective cohort study evaluated immune responses and safety after two doses of CoronaVac in T2D and HCW groups at Chulabhorn Hospital. The levels of total antibodies against the receptor-binding domain (anti-RBD) of the SARS-CoV-2 spike protein at baseline and 4 weeks after vaccination were collected. The level of anti-RBD concentrations was reported as geometric mean concentration (GMC) and compared between groups using the geometric mean ratio (GMR). Results: 81 participants were included; 27 had T2D and 54 were HCW. After complete vaccination, anti-RBD concentrations were not significantly different between T2D (57.68 binding antibody units (BAU)/mL, 95% confidence interval (CI) = 29.08; 114.44) and HCW (72.49 BAU/mL, 95% CI = 55.77; 94.22) groups. Subgroup analysis showed the GMC of anti-RBD was significantly lower in T2D patients with dyslipidaemia (50.04 BAU/mL) than in T2D patients without dyslipidaemia (341.64 BAU/mL). Conclusions: The immune response at 4 weeks after two doses of CoronaVac did not significantly differ between patients with T2D and HCW.

## 1. Introduction

Beginning with the initial outbreak in December 2019, as of February 2022, the cumulative number of confirmed SARS-CoV-2 cases worldwide had reached more than 400 million, with nearly 6 million deaths. The World Health Organization (WHO) named the disease caused by SARS-CoV-2 coronavirus disease 2019 (COVID-19) [1]. Data from several countries in 2020 showed that 14–19% of ill patients required hospitalization, and 3–5% of cases were severe or had complications requiring admission to intensive care units [2].

Diabetes mellitus is one of the underlying noncommunicable diseases that increases the risk for greater severity, more complications and higher mortality associated with COVID-19, likely because patients with diabetes have impaired adaptive and innate immune responses [3].

There are several proposed mechanisms for such complications. One mechanism involves many aspects of white blood cell dysfunction, including neutrophil function and monocyte function, adherence, chemotaxis, phagocytosis, bacterial killing and respiratory burst [4,5,6,7,8,9,10,11,12,13,14]. Other studies supporting this dysfunction found that leukocyte function, granulocyte adherence and phagocytic activity were significantly improved when hyperglycaemia was treated to reduce mean fasting plasma glucose levels [8,11,15,16,17]. Another proposed mechanism in patients with diabetes exposed to transient elevations in glucose involves a rapid reduction in lymphocytes [18]. Hyperglycaemia in diabetic patients was associated with a reduction in T cells, both CD-4 and CD-8 subsets [19]. B- and T-cell responses were compromised and more affected in obese people with type 2 diabetes mellitus [20].

The coronavirus vaccines are important for people with diabetes mellitus to prevent infection and reduce the severity of the infection. Currently, there are several types of coronavirus vaccines, including live-attenuated, inactivated, protein subunit and nucleic acid vaccines. CoronaVac is a Vero-cell-based, aluminium hydroxide-adjuvanted and β-propiolactone-inactivated vaccine [21]. Following the Thailand government’s policy of COVID-19 vaccination in the first phase, a large population in Thailand was vaccinated with CoronaVac. Each 0.5 mL dose of CoronaVac vaccine contains 3 µg of inactivated SARS-CoV-2 virus [22]. The vaccine is intramuscularly administered as two doses, 3 weeks apart (at day 0 and day 21). 

It is established that patients with diabetes have a lower immunity than the general population. However, there are limited data on the immune response to CoronaVac in patients with diabetes. Therefore, this study aimed to assess the antibody response at 4 weeks after the second dose of CoronaVac, by comparing patients with type 2 diabetes and healthcare workers in Thailand.

## 2. Materials and Methods

### 2.1. Study Design and Participants

We conducted a single-centre, age- and sex-matched prospective cohort 1:2 study that evaluated immune responses and safety at 4 weeks after two doses of CoronaVac in patients with type 2 diabetes and healthcare workers at Chulabhorn Hospital. Patients with type 2 diabetes who received CoronaVac were enrolled if they were at least 18 years old. It is important to note that the control group in this study consisted of Chulabhorn Royal Academy employees who were matched in terms of age (within 5 years) and sex to patients with type 2 diabetes. The control group included a range of occupations, such as healthcare providers, healthcare assistants and back-office employees. Importantly, most individuals in the control group had no underlying diseases and were mostly non-diabetic. As such, they may provide a reasonable representation of the general population.

Participants with history of COVID-19 infection, cancer, pregnancy, breastfeeding and history of acute illness or blood transfusion within 90 days were excluded. The study was conducted from 1 June to 31 August 2020. This study was approved by the Ethics Committee for Human Research, Chulabhorn Research Institute. All the patients provided written informed consent before participation. This trial was registered with thaiclinicaltrials.org (TCTR20220720001).

### 2.2. Data Collection and Definitions

Data collection included age, sex, nationality, existing diseases (diabetes mellitus, hypertension, dyslipidaemia, coronary artery disease, chronic kidney disease, end-stage renal disease and cirrhosis) and haemoglobin (Hb) A1C levels in the 90 days before or after, number of current medications for diabetes, insulin use, glucagon-like peptide 1 receptor agonist use, body mass index (BMI) and history of steroid use in the previous 90 days. Diabetes mellitus was defined according to a history of having been diagnosed with diabetes or history of anti-diabetes medication. Hypertension was defined according to systolic blood pressure of ≥140 mmHg or diastolic blood pressure of ≥90 mmHg, or a history of anti-hypertensive therapy. Overweight and obesity were defined according to BMI. Dyslipidaemia was defined as total cholesterol levels ≥ 220 mg/dL or a history of lipid-lowering therapy. Chronic kidney disease and end-stage renal disease were defined according to creatinine clearance or dialysis status. Other diseases were defined by data in medical records.

### 2.3. Procedure

CoronaVac was administered as a 0.5 mL dose intramuscularly injected into the deltoid muscle, with two doses 3 weeks apart (at day 0 and day 21). Reactogenicity was monitored for 30 min after vaccination for immediate events. Thereafter, reactogenicity follow-up was continued at home at days 1 and 7 after vaccination.

A 6 mL blood sample was collected to determine serum levels of the total antibodies against the receptor-binding domain (anti-RBD) of the SARS-CoV-2 spike protein at baseline (before the first vaccine dose), and then 4 weeks after the second dose. Anti-RBD concentrations were measured by an electrochemiluminescence immunoassay Elecsys Anti-SARSCoV-2 S (Elecsys-S) kit (Roche Diagnostics, Mannheim, Germany). 

The range of measurement of anti-RBD antibodies was 0.4–2500 U/mL. The cut-off value for a positive result was >0.8 U/mL, referenced from the manufacturer. Recently, the WHO released an international standard for the measurement of SARS-CoV-2 immunoglobulin that corresponds to the body’s immune response after natural infection or vaccination as binding antibody units (BAU) [23]. Therefore, we used this equation (Elecsys-S U = 0.972 × BAU) to transform Elecsys-S data from U to BAU [24].

### 2.4. Outcomes

Our primary goal was to determine the geometric mean concentration (GMC) of anti-RBD antibodies for SARS-CoV-2 at 4 weeks after two doses of CoronaVac in patients with type 2 diabetes and healthcare workers. Comparison of GMC between groups used the geometric mean ratio (GMR). Secondary outcomes were other factors affecting the GMC of anti-RBD in diabetic patients and adverse events after two doses of vaccine.

### 2.5. Sample Size

A previous study of the ChAdOx1 nCoV-19 (AZD1222) vaccine showed that the GMC of anti-RBD antibodies for SARS-CoV-2 was 15.13 BAU/mL in patients with diabetes versus 40.2 BAU/mL in healthcare personnel. The standard deviation was 52 [25]. The sample size was calculated to achieve a power of 80% with a significance level of 5% (alpha = 0.05). The number of subjects required was expected to be 68. This estimate was based on the assumption that 10% of the participants may be lost to follow-up.

### 2.6. Statistical Analysis

Data entry and analysis were carried out using STATA/SE version 16.1. Continuous variables are shown as the mean ± SD or median and interquartile range. Categorical variables are shown as number and percentage. Anti-RBD antibodies for SARS-CoV-2 were shown as GMC and 95% confidence intervals (CI). Comparison of GMC between groups used multiple linear regression analysis, with *p* < 0.05 indicating statistical significance.

## 3. Results

### 3.1. Baseline Characteristics 

A total of 81 participants were included: 27 patients with type 2 diabetes and 54 healthcare workers. Mean ages were not different between groups: 52.48 and 52.11 years for patients with diabetes and healthcare workers, respectively. Baseline characteristics are described in Table 1. Most of the diabetic cases were obese. The median body weight was 76 kg and median BMI was 28.01 kg/m^2^ in patients with type 2 diabetes. Most healthcare workers were overweight. The median body weight was 65.25 kg and median BMI was 23.61 kg/m^2^. The majority of patients with diabetes had successful glycaemic control, and the mean Hb A1C was 7.04%. Only seven of the diabetic patients (25.94%) had an Hb A1C greater than 8%. The mean number of diabetes drugs used was three. Of the patients with diabetes, four used insulin and others had comorbidities, including dyslipidaemia (92.59%), hypertension (66.67%), coronary artery disease (11.11%), cirrhosis (11.11%) and chronic kidney disease (3.7%). There were no patients positive for human immunodeficiency virus or end-stage kidney disease in this study. One of the patients with diabetes used steroids. The study revealed that a small proportion of healthcare workers (9 out of 54) had comorbidities, with the most prevalent comorbidities being hypertension (present in 14.81% of individuals), dyslipidaemia (present in 9.26% of individuals) and coronary artery disease (present in 1.85% of individuals) (Table 1).

### 3.2. Outcome

#### 3.2.1. Primary Outcome

**Figure 1:**
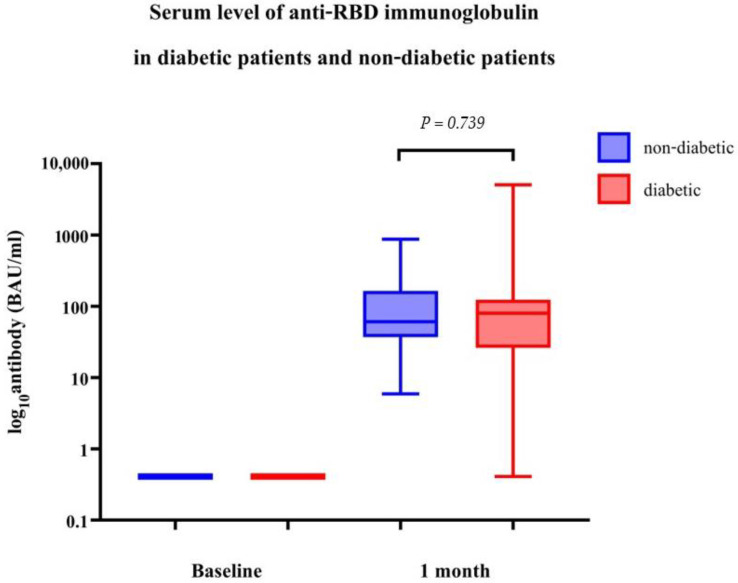
Serum levels of anti-receptor-binding domain (RBD) immunoglobulin in patients with or without diabetes.

Regarding the antibodies present after vaccination, the GMC of anti-RBD antibodies for SARS-CoV-2 at 4 weeks after two doses of CoronaVac was 57.68 BAU/mL (95% CI 29.08; 114.44) and 72.49 BAU/mL (95% CI 55.77; 94.22) in age/sex-matched patients with diabetes and healthcare workers, respectively. The GMR between the groups, adjusted for BMI and comorbidities, was 0.85 (95% CI 0.32; 2.26) and was not significantly different (*p* = 0.739, Table 2 and Figure 1). 

#### 3.2.2. Secondary Outcomes 

A multiple linear regression model was used to compare other factors affecting the GMC of anti-RBD antibodies at 4 weeks after two doses of CoronaVac in patients with diabetes. Potential confounding factors included age, sex, Hb A1C levels, number of current diabetes medications, insulin use, glucagon-like peptide 1 receptor agonist use, BMI, hypertension, dyslipidaemia, coronary artery disease, chronic kidney disease, end-stage renal disease, cirrhosis and steroid use within 90 days (Table 3). Unexpectedly, the factors corresponding to significant differences in anti-RBD antibodies levels in patients with diabetes included dyslipidaemia. The GMC of anti-RBD antibodies was 50.04 and 341.64 BAU/mL in type 2 diabetic patients with or without dyslipidaemia, respectively. The GMR between groups was 0.15 (95% CI 0.07; 0.30), *p* < 0.001. 

Only one case had a history of steroid use, so we could not draw a conclusion about the effect of steroid use. Analysis for other confounding factors found no significant differences in the GMC of anti-RBD antibodies between other subgroups. 

#### 3.2.3. Reactogenicity

**Figure 2:**
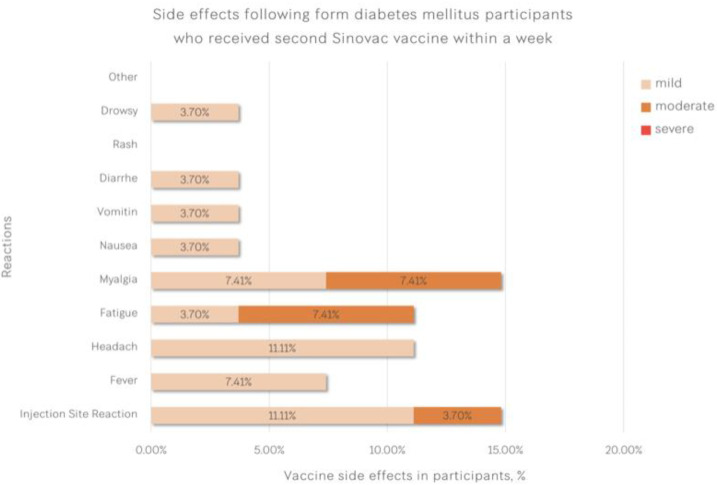
Adverse events after two doses of vaccine in patients with type 2 diabetes.

**Figure 3:**
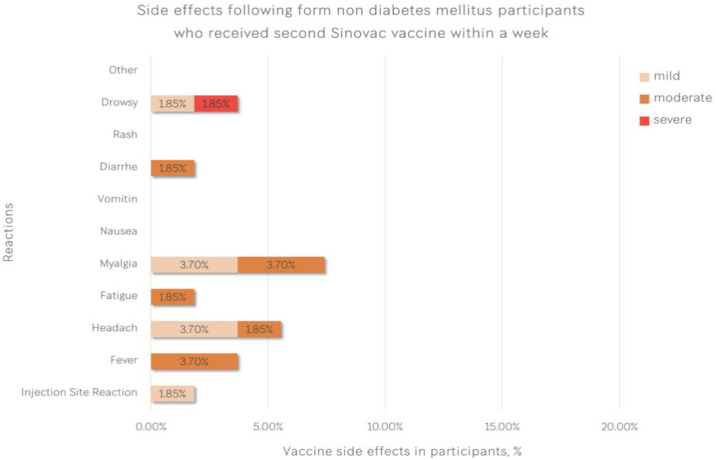
Adverse events after two doses of vaccine in healthcare workers.

Reactogenicities after two doses of vaccine are shown in Table 4. The most common reactogenicities in patients with type 2 diabetes were injection site reaction and myalgia (as shown in Figure 2), whereas the most common adverse events in healthcare workers were myalgia followed by headache (as shown in Figure 3). Four participants with type 2 diabetes (14.8%) and one healthcare worker (1.85%) had injection site reactions (*p* = 0.040). Other adverse events, such as fever, headache and fatigue, were not different between the groups. 

## 4. Discussion

The current study found that 4 weeks after immunization with two doses of CoronaVac, the GMC of anti-RBD antibodies for SARS-CoV-2 in patients with diabetes was not significantly lower than healthcare workers. This result is consistent with previous studies of influenza vaccines, which used inactivated vaccines such as CoronaVac. The antibody responses to an influenza vaccine, as measured by hemagglutination inhibition assays, was similar between well-controlled diabetic elderly and healthy elderly cases [26]. Several previous studies conducted on COVID-19 vaccines’ immunogenicity and effectiveness in patients with diabetes compared to healthy controls. Most of these studies have reported lower vaccine effectiveness and immunogenicity in patients with diabetes when compared to healthy controls [27,28]. However, our study yielded different results, which could be attributed to various factors, such as the type of COVID-19 vaccine used, sample size, levels of glycaemic control and characteristics of the control group. The first factor, vaccine type, is noteworthy, as previous systematic reviews have shown that the majority of COVID-19 vaccines studied are mRNA and recombinant vaccines, while inactivated vaccines comprise a minority. Studies comparing the immunogenicity of different COVID-19 vaccine types have demonstrated that participants who received two doses of Moderna had significantly higher total antibody responses to the receptor-binding domain (RBD) than those who received two doses of AZD1222 (*p* < 0.0001) or two doses of Sinopharm (*p* = 0.03) [29]. These findings align with a systematic review that reported lower efficacy and immunogenicity of inactivated vaccines against COVID-19 infection compared to mRNA and recombinant vaccines [30]. The aforementioned studies have demonstrated that different types of vaccines exhibit varying levels of vaccine effectiveness and immunogenicity. Inactivated vaccines, for instance, have lower immunogenicity and vaccine effectiveness than other vaccines. Consequently, the use of inactivated vaccines in this study could result in a lack of difference in immunogenicity between patients with diabetes and healthcare workers. However, prior research conducted on specific groups, such as those receiving inactivated vaccines for COVID-19, has yielded varying results. For instance, a study examining the CoronaVac and Sinopharm vaccines in patients with diabetes found that anti-RBD-IgG and neutralizing antibodies (Nabs) levels were significantly lower in patients with diabetes (n = 89) than in healthy controls (n = 100) after vaccination [31]. Conversely, another study reported no statistically significant differences in immunogenicity between patients with diabetes and healthy controls. For example, a study on the Vero-cell-derived inactivated COVID-19 vaccine in older patients with hypertension and diabetes mellitus found no statistically significant differences in GMT-neutralizing antibody post-vaccination between groups, including elderly patients with hypertension (n = 325), diabetes (n = 328), combined hypertension and diabetes (n = 292) and healthy controls (n = 468) [32]. Another study involving 76 patients with diabetes (26.4%), who received Pfizer-BioNTech and Sinopharm vaccines and underwent multivariable regression analysis, found no statistically significant negative impact of diabetes mellitus on IgG titre [33].

Hence, it is possible that additional factors may have contributed to the divergent outcomes observed in various studies. One such factor is the level of glycaemic control, which has been shown to impact the immune response. This notion is supported by previous research that demonstrated that diabetic patients with well-controlled blood glucose levels exhibited a more robust immune response following COVID-19 vaccination compared to those with poorly controlled glucose levels [34]. Notably, the majority of the patients with diabetes in our study had well-controlled blood glucose levels, with 60% of them having an HbA1c level of less than or equal to 7%. This may have resulted in no observable differences in the level of immunity between the group of patients with diabetes and healthcare workers.

The characteristics of the control group may be another potential contributing factor. Several studies have demonstrated that body weight and BMI can impact the level of immunogenicity induced by COVID-19 vaccines. For instance, a systematic review and meta-analysis found that obesity was significantly associated with reduced antibody responses to SARS-CoV-2 vaccines, regardless of whether mRNA vaccines, adenovirus vector vaccines or inactivated virus vaccines were used [35]. The findings of a study on inactivated COVID-19 vaccine were consistent with this systematic review and meta-analysis. The study reported that the S-RBD-neutralizing antibody was significantly lower in the BMI >25.00 kg/m^2^ group compared to the 21.00–25.00 kg/m^2^ group (*p* <0.05). Additionally, the S-RBD-neutralizing antibody in both the 21.00–25.00 kg/m^2^ and >25.00 kg/m^2^ groups was significantly lower than that of the ≤21.00 kg/m^2^ group (*p* <0.05) [36]. In our study, the healthcare workers had a median body weight of 65.25 (55–73.5) kg and a median BMI of 23.61 (21.51, 26.24) kg/m^2^, which is considered overweight in the Asian population. Additionally, 18 cases (33.33%) of healthcare workers had an obese BMI considering the average BMI of the Asian population (BMI > 25 kg/m^2^). It is well established that overweight and obesity affect the level of immunogenicity of COVID-19 vaccines, and the high proportion of healthcare workers who met the criteria for Asian obesity may have contributed to impaired immunity. Thus, the difference in anti-RBD antibodies levels between the patients with diabetes and healthcare workers was not significant. 

Another observation we made was that one patient with diabetes had extremely high levels of anti-RBD antibodies (5044.24 MAU/mL) compared to other patients with diabetes, whose geometric mean was 48.57 BAU/mL. This outlier result may have affected the overall analytical results. 

The results of the subgroup analysis in our study show that the geometric mean concentration (GMC) of anti-RBD antibodies in patients with type 2 diabetes and dyslipidaemia was significantly lower than that of diabetic patients without dyslipidaemia. This finding is consistent with a study by Naruse et al. that investigated the BNT162b2 mRNA COVID-19 vaccine [37]. In this study, univariable analysis revealed that anti-RBD IgG levels were significantly lower in patients with cardiovascular disease and dyslipidaemia compared to healthcare workers 14 days after receiving two doses of the BNT162b2 vaccine. This phenomenon can be attributed to the negative effects of dyslipidaemia on the immune system. Studies have shown that dyslipidaemia can have potential effects on both humoral and cellular immunity, leading to immune dysfunction [38,39]. Furthermore, some studies have found that elderly patients on long-term statin therapy have a lower immune response to post-influenza vaccination compared to those not on statin therapy [40]. However, further research may be needed to determine the impact of dyslipidaemia on immune responses to inactivated vaccines.

In this study, only one participant had a history of steroid use, and therefore, it is not possible to draw conclusions about the effect of steroid use on the immune response to the COVID-19 vaccine. Further research with a larger sample size of participants who have a history of steroid use is needed to investigate the impact of steroid use on vaccine efficacy.

With respect to reactogenicity, this study shows that patients with diabetes did not experience significantly different adverse events compared to healthcare workers, except for injection site reactions. This finding aligns with another study that examined the safety and immunogenicity of an inactivated COVID-19 vaccine in older individuals with hypertension and diabetes, which found no significant differences in the incidence of adverse reactions within 21 days after two doses of vaccination across the four groups [32]. Similarly, another study found that within 30 days after vaccination, the overall incidence of adverse events in the type 2 diabetes group and the healthcare workers group did not differ significantly [31]. Therefore, it can be assumed that adverse events after two doses of vaccination were generally similar in type 2 diabetes patients and healthcare workers, with injection site reactions being more common in diabetes patients but potentially occurring incidentally.

Strengths and limitations: The strength of this study was that it focused on patients with diabetes mellitus type 2, which is a significant risk factor for severe COVID-19 infection. Additionally, the study attempted to minimize bias by matching the age/gender variable with the control group. However, the study’s sample size was limited, which may have affected the statistical differences between the two groups. The study was also conducted at a single centre, which may limit the generalizability of the findings to other populations or settings. Another limitation of this study is that neutralizing anti-bodies were not directly measured due to the complexity and limitations of the analysis. Instead, the study used anti-RBD antibodies as a surrogate marker for vaccine effectiveness. Although this approach provided useful information, it may not fully reflect the true level of neutralizing antibodies. To address this limitation, future studies with larger sample sizes and the ability to directly measure neutralizing antibodies may provide more accurate evidence on the vaccine’s effectiveness.

## 5. Conclusions

In conclusion, the GMC of anti-RBD antibodies for SARS-CoV-2 at 4 weeks after two doses of CoronaVac did not significantly differ between patients with type 2 diabetes and healthcare workers.

## Figures and Tables

**Table 1:** Baseline characteristics.

Baseline Characteristics	Type 2 Diabetic Patients	Healthcare Workers	*p*-Value
Age, years (mean ± SD)	52.48 ± 10.71	52.11 ± 10.10	
Male, *n* (%)	13 (48.15)	26 (48.15)	
BW, median (IQR)	76 (60, 86)	65.25 (55–73.5)	0.044
BMI, median (IQR)	28.01 (24.93, 31.45)	23.61 (21.51, 26.24)	<0.001
<25, *n* (%)	3 (11.11)	36 (66.67)	
≥25, *n* (%)	20 (74.07)	18 (33.33)	
Haemoglobin A1C, % (IQR)	7.04 (6.20, 8.10)	NA	
≤6.5, *n* (%)	8 (29.63)	NA	
6.6–7, *n* (%)	8 (29.63)	NA	
7.1–7.9, *n* (%)	4 (14.81)	NA	
≥8, *n* (%)	7 (25.93)	NA	
Number of diabetes medications, % (IQR)	3, (2–4)	NA	
1, *n* (%)	4 (15.38)	NA	
2, *n* (%)	8 (30.77)	NA	
3, *n* (%)	6 (23.08)	NA	
4, *n* (%)	7 (26.92)	NA	
5, *n* (%)	1 (3.85)	NA	
Insulin use, *n* (%)	4 (14.81)	NA	
GLP-1 RA use, *n* (%)	4 (14.81)	NA	
Comorbidities	27 (100.00)	9 (16.67)	<0.001
Hypertension, *n* (%)	18 (66.67)	8 (14.81)	<0.001
Dyslipidaemia, *n* (%)	25 (92.59)	5 (9.26)	<0.001
Coronary artery disease, *n* (%)	3 (11.11)	1 (1.85)	0.105
Chronic kidney disease, *n* (%)	1 (3.70)	0 (0.00)	0.333
Cirrhosis, *n* (%)	3 (11.11)	0 (0.00)	0.034
Steroid use, *n* (%)	1 (3.70)	0 (0.00)	-
End-stage kidney disease, *n* (%)	0 (0.00)	0 (0.00)	1.000
HIV + status *n* (%)	0 (0.00)	0 (0.00)	1.000

BW, body weight; BMI, body mass index; IQR, interquartile range; GLP-1 RA, glucagon-like peptide- 1 receptor agonists; HIV, human immunodeficiency virus; SD, standard deviation.

**Table 2:** Serum levels of anti-RBD immunoglobulin in patients with type 2 diabetes and healthcare workers after vaccination (linear regression, adjusted for BMI, comorbidities).

	Geometric Mean of Anti-RBD Immunoglobulin (BAU/mL) (95% CI)	Geometric Ratio	*p*-Value
DM	57.68 (29.08, 114.44)	0.85 (0.32, 2.26)	0.739
Non-DM	72.49 (55.77, 94.22)	Ref.	

BAU, binding antibody units; CI, confidence interval; DM, diabetes mellitus; RBD, receptor-binding domain.

**Table 3:** Subgroup analysis in patients with type 2 diabetes.

Type 2 Diabetic Patients	Geometric Mean of Anti-RBD Immunoglobulin (BAU/mL) (95% CI)	Geometric Ratio	*p*-Value
Level of HbA1c (%)			
HbA1C ≤ 6.5	48.24 (14.77, 157.49)	Ref.	
HbA1C 6.6–7	30.95 (6.53, 146.75)	0.64 (0.11, 3.63)	0.602
HbA1C 7.1–7.9	56.57 (9.38, 341.12)	1.17 (0.26, 5.35)	0.830
HbA1C ≥ 8	145.82 (20.89, 1017.671)	3.02 (0.43, 21.32)	0.253
Number of DM drugs			
1	83.79 (10.99, 638.89)	Ref.	
2	31.81 (5.25, 192.77)	0.38 (0.35, 3.06)	0.346
3	44.67 (9.26, 215.47)	0.53 (0.09, 3.29)	0.480
4	100.39 (17.41, 578.75)	1.20 (0.16, 8.83)	0.853
5	39.31	0.47 (0.13, 1.69)	0.232
GLP1-RA			
Use	27.39(1.58, 474.19)	0.44 (0.07, 2.77)	0.367
Non-use	62.24 (28.66, 135.18)	Ref.	
Insulin			
Use	63.17 (16.85, 236.82)	1.11 (0.36, 3.40)	0.846
Non-use	56.79 (25.44, 126.75)	Ref.	
BMI (kg/m^2^)			
<25	40.05 (4.99–321.63)	Ref.	
≥25	65.55 (31.58, 136.06)	1.64 (0.26–10.29)	0.585
Age (years)			
18–30	99.96	Ref.	
31–60	52.15 (24.76, 109.82)	0.52 (0.24, 1.11)	0.090
>60	73.72 (5.90, 921.82)	0.74 (0.10, 5.26)	0.752
Sex			
Male	71.01 (24.63, 204.69)	Ref.	
Female	47.57 (17.27, 131.05)	0.67 (1.17, 2.69)	0.558
Comorbidities			
HT			
HT	42.51 (17.75, 101.84)	0.40 (0.10, 1.59)	0.183
Non-HT	106.24 (31.08, 363.16)	Ref.	
DLP			
DLP	50.04 (24.59, 101.83)	0.15 (0.07, 0.30)	<0.001
Non-DLP	341.64 (119.88, 973.58)	Ref.	
CAD			
CAD	11.30 (0.00, 27763.21)	0.16 (0.01, 4.05)	0.253
Non-CAD	70.73 (38.14, 131.20)	Ref.	
Cirrhosis			
Cirrhosis	120.37 (9.48, 1529.01)	2.29 (0.63, 8.28)	0.197
Non-Cirrhosis	52.62 (24.67, 112.25)	Ref.	
CKD			
CKD	100.60	1.78 (0.86, 3.68)	0.114
Non-CKD	56.47 (27.71, 115.06)	Ref.	
Steroid			
Steroid use	0.41	0.01 (0.00, 0.01)	<0.001
Non-steroid use	69.77 (38.84, 125.34)	Ref.	

BAU, binding antibody units; BMI, body mass index; CAD, coronary artery disease; CI, confidence interval; CKD, chronic kidney disease; DLP, dyslipidaemia; DM, diabetes mellitus; GLP-1 RA, glucagon-like peptide-1 receptor agonists; HT, hypertension; RBD, receptor-binding domain.

**Table 4:** Adverse events after two doses of vaccine in patients with type 2 diabetes and healthcare workers.

Reactions	Type 2 Diabetic Patients (%)	Healthcare Workers (%)	*p*-Value
Injection Site Reaction	4 (14.81)	1 (1.85)	0.040
Fever	2 (7.41)	2 (3.70)	0.597
Headache	3 (11.11)	3 (5.56)	0.395
Fatigue	3 (11.11)	1 (1.85)	0.105
Myalgia	4 (14.81)	4 (7.41)	0.431
Nausea	1 (3.70)	0 (0.00)	0.333
Vomiting	1 (3.70)	0 (0.00)	0.333
Diarrhoea	1 (3.70)	1 (1.85)	1.000
Rash	0 (0.00)	0 (0.00)	-
Drowsiness	1 (3.70)	2 (3.70)	1.000
Other	0 (0.00)	0 (0.00)	-

## Data Availability

Not applicable.
